# Correction: Detecting underreporters of abortions and miscarriages in the national study of family growth, 2011–2015

**DOI:** 10.1371/journal.pone.0315112

**Published:** 2024-12-02

**Authors:** Ting Yan, Roger Tourangeau

The phrase “national study of family growth” should have been written as “National Survey of Family Growth” in the article title. The correct title is: Detecting underreporters of abortions and miscarriages in the National Survey of Family Growth, 2011–2015. The correct citation is: Yan T, Tourangeau R (2022) Detecting underreporters of abortions and miscarriages in the National Survey of Family Growth, 2011–2015. PLoS ONE 17(8): e0271288. https://doi.org/10.1371/journal.pone.0271288.[1]
