# Supplementary material for: Detecting underreporters of abortions and miscarriages in the national study of family growth, 2011–2015
Source: PLoS One. 2022 Aug 3;17(8):e0271288. doi: 10.1371/journal.pone.0271288 (PMC9348680; doi:10.1371/journal.pone.0271288)
Supplement: S1 Table — (DOCX) [file pone.0271288.s001.docx]

**S1 Table. Question Wording and Factor Loading for Items in the Traditional Sex Attitudes Scale.**

Question Wording and Factor Loading for Items in the Traditional Sex Attitudes Scale

| Survey Item | Factor Loading |
| --- | --- |
| 1. Sexual relations between two adults of the same sex is all right. (IH-3) 2. It is all right for unmarried 18 year olds to have sexual intercourse if they have strong affection for each other. (IH-5) 3. It is all right for unmarried 16 year olds to have sexual intercourse if they have strong affection for each other. (IH-6) 4. It is okay for young, unmarried woman to have and raise a child. (IH-8) 5. Gay or lesbian adults should have the right to adopt children. (IH-9) 6. A young couple should not live together unless married. (IH-10) 7. It is okay to have and raise children when the parents are living together but not married. (IH-17) 8. Living together before marriage may help prevent divorce. (IH-18) | 0.6848  0.5811  0.4022  0.4868  0.6998  -0.5893  0.5604  0.4467 |
| Variance explained by this factor | 2.6194 |

*Note.* Parenthetical entries are the item numbers. The sixth item (IH-10) was reverse scored. The responses were given on a five-point scale (Agree strongly; Agree; Neither agree nor disagree [if volunteered by the respondent]; Disagree; Disagree strongly).
