# Supplementary material for: Detecting underreporters of abortions and miscarriages in the national study of family growth, 2011–2015
Source: PLoS One. 2022 Aug 3;17(8):e0271288. doi: 10.1371/journal.pone.0271288 (PMC9348680; doi:10.1371/journal.pone.0271288)
Supplement: S2 Table — (DOCX) [file pone.0271288.s002.docx]

**S2 Table. Question Wording and Factor Loading for Items in the Attitudes towards Marriage Scale.**

Question Wording and Factor Loading for Items in the Attitudes toward Marriage Scale

| Survey Item | Factor Loading |
| --- | --- |
| 1. Divorce is usually the best solution when a couple can’t seem to work out their marriage problems. (IH2) 2. People can’t be really happy unless they have children. (IH-6a) 3. Marriage has not worked out for most people I know. (IH-16) | 0.3354  0.2701  0.2086 |
| Variance explained by this factor | 0.7278 |

*Note*. Parenthetical entries are the item numbers. The responses were given on a five-point scale (Agree strongly; Agree; Neither agree nor disagree [if volunteered by the respondent]; Disagree; Disagree strongly).
