# Supplementary material for: Detecting underreporters of abortions and miscarriages in the national study of family growth, 2011–2015
Source: PLoS One. 2022 Aug 3;17(8):e0271288. doi: 10.1371/journal.pone.0271288 (PMC9348680; doi:10.1371/journal.pone.0271288)
Supplement: S3 Table — (DOCX) [file pone.0271288.s003.docx]

|  | 2-indicator LCA Modal Assignment | | | | 2-indicator LCA, Random Assignment | | | | 3-indicator LCA, Modal Assignment | | | | 3-indicator LCA, Random Assignment | | | | Survey Data Only | | | |
| --- | --- | --- | --- | --- | --- | --- | --- | --- | --- | --- | --- | --- | --- | --- | --- | --- | --- | --- | --- | --- |
| Parameter | OR | 95% CI | | *p*-value | OR | 95% CI | | *p*-value | OR | 95% CI | | *p*-value | OR | 95% CI | | *p*-value | OR | 95% CI | | *p*-  value |
| Age (centered at mean) | **1.08** | 1.03 | 1.13 | <0.01 | **1.07** | 1.02 | 1.12 | 0.01 | **1.10** | 1.05 | 1.15 | <0.01 | **1.11** | 1.06 | 1.17 | <.0001 | **1.08** | 1.03 | 1.13 | <0.01 |
| Married or Cohabitating | 1.16 | 0.70 | 1.94 | 0.56 | 1.19 | 0.73 | 1.93 | 0.48 | 1.44 | 0.85 | 2.45 | 0.17 | 1.63 | 0.97 | 2.76 | 0.07 | 1.16 | 0.70 | 1.94 | 0.56 |
| Hispanic or Black | **0.58** | 0.34 | 0.98 | 0.04 | 0.61 | 0.35 | 1.06 | 0.08 | 0.69 | 0.39 | 1.23 | 0.20 | 0.64 | 0.34 | 1.19 | 0.15 | **0.58** | 0.34 | 0.98 | 0.04 |
| No Children in Household | 0.77 | 0.45 | 1.30 | 0.32 | 0.74 | 0.44 | 1.24 | 0.25 | 0.60 | 0.34 | 1.08 | 0.09 | **0.54** | 0.30 | 0.96 | 0.04 | 0.77 | 0.45 | 1.30 | 0.32 |
| Number of Pregnancies | 0.91 | 0.78 | 1.05 | 0.17 | 0.86 | 0.74 | 1.00 | 0.06 | 0.92 | 0.81 | 1.06 | 0.26 | 0.90 | 0.78 | 1.03 | 0.13 | 0.91 | 0.78 | 1.05 | 0.17 |
| Number of Life Partners | 0.99 | 0.97 | 1.02 | 0.65 | 0.99 | 0.96 | 1.02 | 0.48 | 1.01 | 0.98 | 1.04 | 0.74 | 1.01 | 0.98 | 1.04 | 0.66 | 0.99 | 0.97 | 1.02 | 0.65 |
| No Religion | **0.46** | 0.29 | 0.73 | <0.01 | **0.40** | 0.25 | 0.63 | <0.01 | **0.57** | 0.36 | 0.91 | 0.02 | **0.61** | 0.38 | 0.99 | 0.04 | **0.46** | 0.29 | 0.73 | <0.01 |
| Total Income | **0.92** | 0.86 | 0.97 | <0.01 | **0.92** | 0.87 | 0.98 | 0.01 | **0.91** | 0.85 | 0.96 | 0.00 | **0.91** | 0.85 | 0.97 | 0.00 | **0.92** | 0.86 | 0.97 | <0.01 |
| Metropolitan Area | 0.60 | 0.34 | 1.05 | 0.07 | 0.60 | 0.35 | 1.04 | 0.07 | 0.58 | 0.33 | 1.01 | 0.06 | 0.57 | 0.32 | 1.02 | 0.06 | 0.60 | 0.34 | 1.05 | 0.07 |
| Mother with High School Education or Less | 1.01 | 0.62 | 1.67 | 0.96 | 1.06 | 0.64 | 1.75 | 0.82 | 1.03 | 0.58 | 1.82 | 0.92 | 1.09 | 0.62 | 1.92 | 0.77 | 1.01 | 0.62 | 1.67 | 0.96 |
| Born outside USA | 1.09 | 0.48 | 2.43 | 0.84 | 0.96 | 0.41 | 2.27 | 0.93 | 0.94 | 0.48 | 1.85 | 0.85 | 0.81 | 0.38 | 1.72 | 0.58 | 1.09 | 0.48 | 2.43 | 0.84 |
| Interview Language | 1.62 | 0.41 | 6.46 | 0.49 | 1.54 | 0.38 | 6.17 | 0.54 | 1.35 | 0.34 | 5.29 | 0.66 | 1.26 | 0.30 | 5.36 | 0.75 | 1.62 | 0.41 | 6.46 | 0.49 |
| Risky Substance Use Behaviors | 0.78 | 0.57 | 1.06 | 0.11 | **0.70** | 0.50 | 0.98 | 0.04 | **0.74** | 0.56 | 0.98 | 0.03 | **0.69** | 0.52 | 0.93 | 0.01 | 0.78 | 0.57 | 1.06 | 0.11 |
| Traditional Sexual Attitudes | **1.48** | 1.05 | 2.08 | 0.03 | 1.34 | 0.95 | 1.90 | 0.10 | **1.57** | 1.09 | 2.25 | 0.02 | **1.51** | 1.05 | 2.16 | 0.03 | **1.48** | 1.05 | 2.08 | 0.03 |
| Attitudes toward Marriage | 1.04 | 0.76 | 1.42 | 0.80 | 1.01 | 0.72 | 1.41 | 0.96 | 0.98 | 0.69 | 1.39 | 0.92 | 0.93 | 0.63 | 1.37 | 0.72 | 1.04 | 0.76 | 1.42 | 0.80 |
| n | 696 | | | | 702 | | | | 678 | | | | 665 | | | | 696 | | | |
| Pseudo R-Square | 0.1846 | | | | 0.1867 | | | | 0.2146 | | | | 0.2301 | | | | 0.1846 | | | |

**S-3 Table: Results of Logistic Regression Models Predicting Underreporting of Abortion**
