# Supplementary material for: Detecting underreporters of abortions and miscarriages in the national study of family growth, 2011–2015
Source: PLoS One. 2022 Aug 3;17(8):e0271288. doi: 10.1371/journal.pone.0271288 (PMC9348680; doi:10.1371/journal.pone.0271288)
Supplement: S4 Table — (DOCX) [file pone.0271288.s004.docx]

|  | 2-indicator LCA Modal Assignment | | | | 2-indicator LCA, Random Assignment | | | | 3-indicator LCA, Modal Assignment | | | | 3-indicator LCA, Random Assignment | | | | Survey Data Only | | | |
| --- | --- | --- | --- | --- | --- | --- | --- | --- | --- | --- | --- | --- | --- | --- | --- | --- | --- | --- | --- | --- |
| Parameter | OR | 95% CI | | *p*-value | OR | 95% CI | | *p*-value | OR | 95% CI | | *p*-value | OR | 95% CI | | *p*-value | OR | 95% CI | | *p*-value |
| Age (centered at mean) | **1.06** | 1.03 | 1.09 | <0.001 | **1.06** | 1.02 | 1.09 | <0.01 | **1.07** | 1.04 | 1.11 | <.0001 | **1.08** | 1.05 | 1.11 | <.0001 | **1.06** | 1.03 | 1.09 | <0.001 |
| Married or Cohabitating | **0.63** | 0.42 | 0.94 | 0.03 | 0.75 | 0.50 | 1.13 | 0.17 | 0.77 | 0.52 | 1.15 | 0.20 | 0.84 | 0.55 | 1.27 | 0.39 | **0.63** | 0.42 | 0.94 | 0.03 |
| Hispanic or Black | **1.53** | 1.07 | 2.20 | 0.02 | **1.70** | 1.17 | 2.47 | 0.01 | **1.68** | 1.18 | 2.40 | <0.01 | **1.71** | 1.21 | 2.43 | <0.01 | **1.53** | 1.07 | 2.20 | 0.02 |
| No Children in Household | **0.50** | 0.32 | 0.79 | <0.01 | **0.55** | 0.35 | 0.85 | 0.01 | **0.40** | 0.25 | 0.66 | <0.001 | **0.35** | 0.21 | 0.59 | <.0001 | **0.50** | 0.32 | 0.79 | <0.01 |
| Number of Pregnancies | **0.81** | 0.72 | 0.92 | <0.01 | **0.77** | 0.67 | 0.89 | <0.001 | **0.84** | 0.75 | 0.95 | <0.01 | **0.79** | 0.70 | 0.90 | <0.01 | **0.81** | 0.72 | 0.92 | <0.01 |
| Number of Life Partners | 0.98 | 0.96 | 1.01 | 0.12 | 0.98 | 0.96 | 1.01 | 0.12 | 0.99 | 0.97 | 1.01 | 0.30 | 0.99 | 0.97 | 1.01 | 0.47 | 0.98 | 0.96 | 1.01 | 0.12 |
| No Religion | 0.94 | 0.61 | 1.45 | 0.78 | 0.96 | 0.62 | 1.48 | 0.84 | 0.98 | 0.63 | 1.52 | 0.92 | 1.02 | 0.66 | 1.59 | 0.91 | 0.94 | 0.61 | 1.45 | 0.78 |
| Total Income | **0.94** | 0.90 | 0.97 | <0.01 | **0.94** | 0.91 | 0.98 | <0.01 | **0.93** | 0.90 | 0.97 | <0.001 | **0.93** | 0.89 | 0.97 | <0.001 | **0.94** | 0.90 | 0.97 | <0.01 |
| Metropolitan Area | 1.34 | 0.93 | 1.92 | 0.11 | 1.26 | 0.87 | 1.82 | 0.22 | 1.29 | 0.91 | 1.85 | 0.16 | 1.30 | 0.89 | 1.91 | 0.17 | 1.34 | 0.93 | 1.92 | 0.11 |
| Mother with High School Education or Less | 1.06 | 0.75 | 1.50 | 0.76 | 1.15 | 0.80 | 1.65 | 0.45 | 1.02 | 0.72 | 1.45 | 0.91 | 1.13 | 0.79 | 1.62 | 0.49 | 1.06 | 0.75 | 1.50 | 0.76 |
| Born outside USA | 1.10 | 0.65 | 1.85 | 0.73 | 1.06 | 0.64 | 1.76 | 0.82 | 1.10 | 0.64 | 1.90 | 0.73 | 1.08 | 0.64 | 1.83 | 0.76 | 1.10 | 0.65 | 1.85 | 0.73 |
| Interview Language | 1.10 | 0.48 | 2.51 | 0.82 | 1.00 | 0.42 | 2.36 | 1.00 | 0.97 | 0.41 | 2.26 | 0.93 | 0.90 | 0.37 | 2.19 | 0.82 | 1.10 | 0.48 | 2.51 | 0.82 |
| Risky Substance Use Behaviors | 1.14 | 0.92 | 1.41 | 0.24 | 1.06 | 0.86 | 1.31 | 0.60 | 1.11 | 0.89 | 1.37 | 0.35 | 1.12 | 0.91 | 1.37 | 0.30 | 1.14 | 0.92 | 1.41 | 0.24 |
| Traditional Sexual Attitudes | 1.01 | 0.79 | 1.28 | 0.94 | 0.95 | 0.75 | 1.21 | 0.66 | 0.99 | 0.78 | 1.27 | 0.95 | 0.94 | 0.73 | 1.21 | 0.62 | 1.01 | 0.79 | 1.28 | 0.94 |
| Attitudes toward Marriage | **0.71** | 0.55 | 0.92 | 0.01 | **0.70** | 0.54 | 0.90 | 0.01 | **0.69** | 0.53 | 0.89 | 0.01 | **0.68** | 0.52 | 0.89 | 0.01 | **0.71** | 0.55 | 0.92 | 0.01 |
| n | 1351 | | | | 1316 | | | | 1330 | | | | 1330 | | | | 1351 | | | |
| Pseudo R-Square | 0.0992 | | | | 0.0973 | | | | 0.1051 | | | | 0.1164 | | | | 0.0992 | | | |

**S-4 Table: Results of Logistic Regression Models Predicting Underreporting of Miscarriage**
