# Supplementary material for: Detecting underreporters of abortions and miscarriages in the national study of family growth, 2011–2015
Source: PLoS One. 2022 Aug 3;17(8):e0271288. doi: 10.1371/journal.pone.0271288 (PMC9348680; doi:10.1371/journal.pone.0271288)
Supplement: Attachment — Submitted filename: PLOS_Rebuttal_letter_04182022.docx [file pone.0271288.s005.docx]

Dear Editor,

We have revised our manuscript to address comments from reviewers. Below we explained how we addressed each comment. We hope that this revision is satisfactory to you.

Best Regard

Ting Yan and Roger Tourangeau

**Journal Requirements:**

1. Please ensure that your manuscript meets PLOS ONE's style requirements, including those for file naming. The PLOS ONE style templates can be found at 
[https://journals.plos.org/plosone/s/file?id=wjVg/PLOSOne_formatting_sample_main_body.pdf](https://secure-web.cisco.com/1vbFUpFWBhomyXjZrubW7eLoLrRdHAP3g04mv-4PAx4wPpzFnzjgAZ0aAuOqhvXoopgw1sT6IZcIIa_4ogzZAY8_VQFomR__mARSGkc_Y0nNMw57sS9gAZZWcCtilrwrKBcgFb3fG3lMoPoRuCKUZLt9fwEFkB974qLBCEg82Z7K_oE7oah3cAT_QcwkVA2NNl9gKXzEZA6JQhQIc9TaEtYt_haR2YMutXp8lx5AqRyMXMM9DosMZaqUzo2g6MZkStmKW7FTMdEAwGr9veQ5VzQ/https%3A%2F%2Fjournals.plos.org%2Fplosone%2Fs%2Ffile%3Fid%3DwjVg%2FPLOSOne_formatting_sample_main_body.pdf) and 
[https://journals.plos.org/plosone/s/file?id=ba62/PLOSOne_formatting_sample_title_authors_affiliations.pdf](https://secure-web.cisco.com/1CFBu5DdaS1JDB3pAL2wqeNO0cr66L_3c5sL9pMPIcxHBNnrCoUS6aMhGEgkSxtbSd6PPu-VfPce5LF7QyNtHMLFYZVnFu-k8O-IKrhz2PASV_nFPUVlytiT07ezLSsFnLeEL2SieVkgrcWcTrtPUBnMU7bFlMYWjpPvqTtxjp6tUfXKWLPPCGzxJGQJ6M2qLI9itfzDA_BVzmUHbdaXpGS88cabPMf-cHIMIrRtXBn3LoBhg4SmyF43szVOB51-Uzrui9BeW1WuvBMJsQPyNVQ/https%3A%2F%2Fjournals.plos.org%2Fplosone%2Fs%2Ffile%3Fid%3Dba62%2FPLOSOne_formatting_sample_title_authors_affiliations.pdf)

*-We made sure that our revised manuscript meets PLOS ONE’s style requirements.*

2. Thank you for stating the following financial disclosure:

The work reported here was supported by a grant (R01HD084473) from the Eunice Kennedy Shriver National Institute of Child Health & Human Development to The Guttmacher Institute (Laura Lindberg, PI). The content is solely the responsibility of the authors and does not necessarily represent the official views of the National Institutes of Health.

Please state what role the funders took in the study.  If the funders had no role, please state: "The funders had no role in study design, data collection and analysis, decision to publish, or preparation of the manuscript." 
If this statement is not correct you must amend it as needed. 
Please include this amended Role of Funder statement in your cover letter; we will change the online submission form on your behalf.

*-We’ve made the required changes and included the amended Role of Funder Statement in our cover letter.*

3. Thank you for stating the following in the Acknowledgments Section of your manuscript:

The work reported here was supported by a grant (R01HD084473) from the Eunice Kennedy Shriver National Institute of Child Health & Human Development to The Guttmacher Institute (Laura Lindberg, PI). The content is solely the responsibility of the authors and does not necessarily represent the official views of the National Institutes of Health.

Please note that funding information should not appear in the Acknowledgments section or other areas of your manuscript. We will only publish funding information present in the Funding Statement section of the online submission form. 
Please remove any funding-related text from the manuscript and let us know how you would like to update your Funding Statement. Currently, your Funding Statement reads as follows:

The work reported here was supported by a grant (R01HD084473) from the Eunice Kennedy Shriver National Institute of Child Health & Human Development to The Guttmacher Institute (Laura Lindberg, PI). The content is solely the responsibility of the authors and does not necessarily represent the official views of the National Institutes of Health.

Please include your amended statements within your cover letter; we will change the online submission form on your behalf.

*-We’ve made the required changes and included the amended statements in our cover letter.*

4. Thank you for stating the following in your Competing Interests section:

No authors have competing interests

Please complete your Competing Interests on the online submission form to state any Competing Interests. If you have no competing interests, please state "The authors have declared that no competing interests exist.", as detailed online in our guide for authors at [http://journals.plos.org/plosone/s/submit-now](http://secure-web.cisco.com/1b6VQTTqGzHEslemDDFdHETmg2OT5B9gfJ_ROSmZXZJ-jDRdB-PBW2EyvoUvyOT4OSSVGQ2l6NnvgzdaPyDyZWjqR-Mtl8Vl65Wa1-6qFat2A_koDl31IpX4IVDnA2YQeB6zEUWZbUquBTLkWn1O2yRthRq_UhFPU-52vrxE5j8OqFN9juJZXINwN0PirqD4G_rVX-dZrwHYSNczAtJb7X1cNqB7SzgXx6492v2wYQJKbjEZA6O8IK-qAXHavkO1OhEpCJ987QbxTnsVBdDvp0w/http%3A%2F%2Fjournals.plos.org%2Fplosone%2Fs%2Fsubmit-now) 
 This information should be included in your cover letter; we will change the online submission form on your behalf.

*-We’ve made the required changes and included the amended statement in our cover letter.*

**Reviewers' comments:**

**Reviewer #1**:

-Although the topics under study are of importance and the paper makes advances in the methodological issues with measurement of these topics, the paper has two central weaknesses that limit it from fully or convincingly reaching its high potential. First, the paper is confusingly framed and situated, especially as it relates to the inclusion of the analyses related to abortion. For example, a great deal is known about the characteristics of women who underreport abortions, including specifically in the NSFG as the paper cites in the introduction. Yet, the paper motivates its analysis by suggesting little is known about the characteristics of these women. While the paper uses individual-level data, which allows for the analysis of new or additional characteristics associated with underreporting, this nuance is not clear in their introduction. More generally, the introduction and background sections of the paper are disconnected and difficult to follow (specific comments related to this can be found in the minor or stylistic issues section following these comments). Relatedly, it is unclear why the paper addresses both abortion and miscarriage underreporting. As aforementioned, abortion underreporting is well-documented, especially in the NSFG. The analysis of miscarriages, however, is quite novel. Thus, the framing of the paper is confusing and does not seem to adequately review the literature related to abortion underreporting nor make a convincing argument or present a clear rationale for why these issues are discussed in-tandem in the paper.

*-We revised the framing of the paper by substantially revising the Introduction. We also made it clear in the Introduction that the paper uses individual-level data and examines the impact of attitudes on underreporting of abortion and miscarriage.*

Second, the paper oversells the meaning of the differences between the CAPI and ACASI comparisons. That is, the paper suggests that inconsistent reporting is equivalent to underreporting, yet, there may well be something substantively different between women who never report an abortion they have had and those who sometimes will report it (on CAPI but not ACASI). There seems to be no way to know if these women are qualitatively similar or if we can draw conclusions about under-reporters as an entire group based on these inconsistent reporters. This approach is novel and interesting, but it seems to fall especially short in the case of abortion where external estimates to quantify underreporting exist. In other words, if the paper were solely focused on miscarriage, it would be more methodologically justifiable to use this approach (since a better alternative is currently lacking). Additionally, the comparison of the LCA to the inconsistent reporters is also less compelling considering these issues.

*-We were* ***not*** *trying to oversell the meaning of the differences between the CAPI and ACASI comparisons. As a matter of a fact, we agreed with the reviewer and acknowledged it as a limitation for using only survey data to investigate abortion and miscarriage underreporting (Lines 72-73; Lines 435-436). The purpose of using LCA in this paper is to find an alternative method to identify underreporters in a realistic setting when no medical records are available and when we do not have to rely on the differences between CAPI and ASCAI.*

In addition to the more major issues outlined above, I also provide feedback on minor or stylistic issues by section below.
Introduction
- It is not clear why the section on LCA is presented in the introduction/background section. It seems that the materials here would be more appropriate in the analytic method section, and the limitations of LCA could be explored in the discussion section.

*-We moved the section on LCA to the analytic method section and moved the limitations of LCA to the discussion section.*

- The structure of the introduction could be revised for clarity as well as to more specifically discuss underreporting of abortion and miscarriages.

*-We revised the structure of the introduction and hope that it is clearer.*

Methods
- It would be more accurate to label “truthful” reporter “consistent” reporters While it is unlikely someone would report an abortion or miscarriage they didn’t have, consistent reporters more accurately describes what is being observed (consistency between CAPI and ACASI)

*-If we had only relied on survey data in our analyses, we agreed with Reviewer 1 that “consistent” reporters would be more accurate. However, we went beyond consistency between CAPI and ACASI; we used “truthful” reporters to refer to women who were classified as “had an abortion (or a miscarriage)” by LCA and who indeed did report having had an abortion (a miscarriage) in both modes. This paper focused on the concordance between the latent class women respondents were assigned to and the answers they provided.*

- The choice to collapse Black and Hispanic populations together should be explicated, especially considering the import of language and religiosity in the findings. It would be stronger to separate these groups, if there is sufficient power

-*We reran logistic regression analyses having Hispanic and Black populations as separate variables. The model results showed that Black women and Hispanic women were less likely to underreport abortion (and more likely to overreport miscarriage) than white women. However, the associations were no longer statistically significant due to insufficient power. Having separate Black and Hispanic groups did not change the size and significance level of other associations in the models. As a result, we kept the original models that used the collapsed category.*

Results
- More descriptive table names would be helpful, especially for Table 5

-*We revised the table names.*

- Person-first or person-centered language should be used (i.e., Black women or Black respondents or respondents of color, etc. rather than “Blacks”)

-*We made the requested changes where is appropriate.*

Discussion
- This section would be stronger with less reiteration of the findings and more exploration of the patterns observed and contribution

-*We revised this section to reduce repetitions and added more exploration.*

- It seems out of place to have a reference to a table in the discussion

*-We removed the reference to the table in the discussion*

- Inclusion of citations and references to work focused on how to improve abortion reporting or reporting of sensitive questions would be useful in the discussion

-*We included citations and reference on work to improve abortion reporting or reporting of sensitive questions.*

**Reviewer #2:**

The utility of latent class analysis method, which is a powerful method for unobservable latent constructs, provides a new insight to classify respondents into “underreporter” and “reporter” groups. I think, main novelty of the study is to test utility of LCA method when estimating accurate abortion reports in a comparative way with survey reports, without any need to medical records. As you have stated in your paper, underreporting of abortions within the scope of mode differences is well-studied issue for NSFG data. However, constructing new scales on sexual attitudes and attitudes towards marriage contributes to current literature on determining factors behind underreporting behaviors of respondents.

So, I recommend revisions based on the following statements:

1- I suggest more specific manuscript title in accordance with study interests:
“Detecting Underreporters of Abortions and Miscarriages in the U.S.: A Latent Class Analysis Approach from NSFG, 2011-2015”.

*-We like the suggestion and adopted the title. We made two minor edits though: 1) we dropped the “latent class analysis approach” since we used two different methods to detect underreporters; 2) we fully specified the acronym “NSFG”.*

2- Although the paper focuses on underreporting of abortions and miscarriages in the U.S., it is also a significant issue for other country settings. Moreover, various methodological approaches were tried to detect underreporters, and calculate accurate abortion rates at the end.

I suggest some recent works for background section. These studies include new techniques to understand underreporting for the U.S. and other countries, as well as characteristics of underreporters.

Lara, D., Strickler, J., Olavarrieta, C.D., & Ellertson, C. (2004). Measuring induced abortion in Mexico: a comparison of four methodologies. Sociological Methods & Research 32(4), 529–558.
Saraç, M., & Koç, İ. (2019). Increasing misreporting levels of induced abortion in Turkey: is this due to social desirability bias?. Journal of Biosocial Science, 52(2), 213-229.
Anderson, B.A., Katus, K., Puur, A. & Silver, B.D. (1994). The validity of survey responses on abortion: evidence from Estonia. Demography 31(1), 115–132
Tennekoon, V. S. (2017). Counting unreported abortions: A binomial-thinned zero-inflated Poisson model. Demographic Research, 36, 41-72.
Medeiros M & Diniz D (2012) Recommendations for abortion surveys using the ballot-box technique. Ciência & Saúde Coletiva 17(7), 1721–1724.

-*Thank you for the suggestions. We added them to the manuscript.*

3- I suggest adding ‘abortion’, ‘miscarriage’, and ‘NSFG’ to the keywords.

*-Thank you for the suggestion. We added these keywords.*

4- Line 35: The authors stated that abortion is more stigmatized than miscarriage. There might be some cultural and political reasons behind that argument. The authors should provide justifications/works for that argument.

*-We used the findings from study [11] to justify the statement that abortion is more stigmatized than miscarriage.*

5- Line 64: ..... to understand why abortions are so badly underreported in surveys by..... Instead of that, I recommend ..... to understand factors affecting underreporting behavior of respondents by identifying the characteristics .... I think, this is more appropriate compared to “why” question, when the authors’ statistical analyses are considered.

*-Thank you for the suggestion. We made the change.*

6- Starting from Line 64: A bit difficult to follow study aims. The authors should re-organize study goals as the following. My suggestion is also more appropriate for the flow of sections about analytical strategy and results.

First goal: Evaluating utility of the LCA models in identifying underreporters of abortions/miscarriages.
Second goal: Investigating characteristics of underreporters of abortions/miscarriages.
Third goal: Determining common and differential features of respondents who underreport their abortions and miscarriages.

*-Thank you for the suggestions. We made the changes and reorganized the goals.*

7- Line 124: For the readers, the authors should add a footnote for explanations of false positive and false negative errors.

*-Thank you for the suggestion. We added a footnote on p9 to explain false positive and false negative errors.*

8- Line 132: For a two-class LCA, three indicators are necessary for the model to be identification. The authors should add a reference here for required number of indicators to achieve model identification.

*-A citation was added to that statement.*

9- Line 173: … other variables. The authors should clarify on which stratification variables were used in the NSFG’s sample design, except for census division and population parameters?

*-We clarified the stratification variables.*

10- Line 182: …. selecting one of the eligible persons for the main interview. The authors should clarify on which selection method were used to select an eligible person to interview? (Kish method, birthday method or else?)

-*It is just a random selection with differential selection probabilities assigned to different age (15 to 19 vs 20 to 44) and sex combinations.*

11- Line 191: Author should give a brief information about sensitive questions in ACASI part of the questionnaire (except for abortion and miscarriage), to give a general picture for readers.

-*We provided a few examples of sensitive questions included in the ACASI part of the questionnaire (Line 128).*

12- The authors defined “underreporter”, if a respondent who reported abortion/miscarriage in only one mode (regardless of its type; CAPI or ACASI). Instead, in accordance with the current literature, I am suggesting to define “underreporter”, if a respondent reported abortion in ACASI mode, but at the same time did not report it in CAPI mode.

- *“Underreporter” is defined as someone who reported having had an abortion in ACASI, but not in CAPI for analyses involving survey data only. For analyses involving LCA, “underreporter” is defined as women who were classified as “had an abortion (or a miscarriage)” by LCA but who did not report having had an abortion (or a miscarriage) in one of the modes.*

As the authors discussed comprehensively in background section, respondents tend to underreport sensitive issues in face-to-face modes (i.e. CAPI) compared to self-administered modes (i.e. ACASI).

Replicating same analyses for that sub-group of respondents (ACASI reporter, CAPI underreporter) and re-organizing the paper would be useful. It is also more appropriate for the current literature and structural integrity of the paper.

*-The logistic regression models using survey data only examined characteristics of women who reported abortion/miscarriage in ACASI but not in CAPI.*

13- Line 232: We used PROC LCA in SAS to fit the models.
Were the complex sample design features adjusted into LCA models?, likewise binary logistic regression analysis mentioned in Line 282. If yes, the authors should give information about that procedure, too. If no, I suggest incorporating weight, psu, and stratification variables into PROC LCA procedure in SAS.

*-Our purpose of using LCA is to generate posterior probabilities, which are subsequently used to assign women respondents into one of the two latent classes. We do not intend to make inferences, as a result, we did not account for clustering and weights when using PROC LCA.*

*We did incorporate clustering, stratification, and weights into logistic regression models predicting women underreporting abortion and miscarriage.*

14- Line 234: …these assignments were based on posterior class membership probabilities. This statement should be excluded. Because it has already stated within second method (Line 239).

-*We removed the sentence.*

15- Line 241: … we divided the respondent into four groups…
I suggest moving text into an organised scheme that is prepared to classify these sub-groups. It would be more useful for readers. In this scheme, the authors should provide unweighted case numbers for women groups.

*-The unweighted case count for each group is provided in Table 3 for abortion and Table 5 for miscarriage.*

16- Line 300: I guess, referring Table 2 should be replaced with Table 1 that includes survey reports.

*-We fixed the typo.*

17- Line 330: The two different methods of identifying underreporters of abortion produce very similar results. This result shows us validity/success of LCA models, authors should a sentence about that here.

*-The LCA results are similar to the results based on the survey data alone. It seems to us that the LCA did not bring much advantage in identifying underreporters. As a result, we are not sure we regard the LCA as a success.*

18- Table 4 should include a note for representation of significance levels, as the authors added for Table 6.

*-We added the note for representation of significance levels.*

19- In my opinion, results about live births should be excluded from Table 5 and scope of the paper. Abortions and miscarriages are quite relevant with literature and authors’ study objectives. There is no need to mention about live births under the results of miscarriages. Thus, I recommend restrict Table 5 only with miscarriages.

*-We agree and now dropped the results about live births.*

20- Line 374-377: Authors should add more discussions about the results on differential characteristics between underreporters of miscarriages and abortion. Why minority groups and marital status produce different results for two groups? Authors may refer evidence from literature here. It is also valid for discussion section (Line 428-434), too. Adding a brief explanation would be useful.

*- The results for marital status are nonsignificant for abortion reporting and weak for miscarriage reporting. We are not sure there is a real difference here. We added something about the difference for marital status (see line 407-410)*

21- At the end of the discussion section, I would like to see three points:
-Can LCA method be an alternative way for other surveys in countries where there are no available medical records or, when there is only one mode of data collection?
-Could authors suggest any practical implications based on study results, especially when underreporters’ characteristics are considered? What can authors suggest for survey stages (e.g. questionnaire design, interviewer training, and data collection).
-To light future studies, which topics related to survey research are suggested by authors including use of LCA models?

*- We added some language at the very end of the section (lines 451-454).*

22- Authors should provide exploratory factor analysis results to construct scales (like S1 Table and S2 table). The loadings of items under extracted factors and explained variances of factors should be provided in that table so that readers could follow results easily.

*- We added factor loadings and the explained variances to S1 and S2 tables.*

Reviewer #3: Broad reservation:
There is a tension in the article regarding its purpose.
The abstract and the paper look different. The abstract is focused on having two different measures of abortion and miscarriage and evaluating their consistency. This is interesting in itself and can be done focusing only on these two evaluations (and possibly analyzing with regression or means, who are the “inconsistent” reporters).
The paper is an application of latent class analysis that, in my opinion, is more debatable. Latent class analysis is about finding groups in the data, but these groups have no name. You do not get a class of “Had an abortion in last five years” vs “Did not have an abortion in last five years” as in table 2. That is pushing the method too much. In particular, when you need to introduce third unrelated variables for identification. If you bring marital status, as you do for instance, the two groups would be people who tend to have one of the marital statuses and report abortion or miscarriage in some of the dimensions and another that does not. This is different from the interpretation that I quoted. I think the paper is good while it develops the analysis in table 1, but the application of LCA is more troublesome and its interpretation should be much more cautious. I would redo or remove the LCA part.

-*We agree with Reviewer 3 that latent class analysis is about finding groups (that is, the latent classes) in the data and we agree that LCA does not give a name to each latent class. However, we respectfully disagree with Reviewer 3 and believe that we can make sense of each latent class and label it accordingly. It is the same as how factors extracted from a factor analysis are interpreted and labeled. Latent class analysis is essentially a factor analysis for categorical variables. We do not think that we’ve pushed the method too much by interpreting and labeling each latent class. It is a typical procedure carried out by applications of LCA in the survey context (e.g., Biemer and Wiesen 2002; Biemer and Witt 1996; Kreuter, Yan, and Tourangeau 2008; Yan, Kreuter, and Tourangeau 2012).*

You should think what is the purpose of the article. If you think what the abstract says is the relevant part, the article should not be as it is.

*-We’ve revised the abstract and the framing of the paper.*

Other comments:
Based on the reservation stated above, and independently of where the article heads for, the abstract needs to be rewritten, perhaps a structured format will help. Currently, it does not talk about the results or implications of the study.

*-We’ve revised the abstract.*

Missing recent references on underreporting in NSFG such as
Desai, S., Lindberg, L.D., Maddow-Zimet, I. et al. The Impact of Abortion Underreporting on Pregnancy Data and Related Research. Matern Child Health J 25, 1187–1192 (2021). [https://doi.org/10.1007/s10995-021-03157-9](https://secure-web.cisco.com/14Fj3Pd51WiZ31sQFinysk9eCHPtnvExqWzs03oN-520pjOcrBkK_78zA3ADSg_uQVYQt1Vz4lDdXge5FTNMmuCKyccmnME5ROUJ9KCr3WF8fpaGgym68ztq_91UXcPczEyIALugLwDqQFKsimTTAUdtKaxghws_EljJTx6qpBSGCNawivduLfm3G8LLioFW5MwHi5Vy7t-9rMYOarkTGpEqEdwiddhNw8byBjrhds51YajGe9C6qT3IyuHxBF2TCnOc74jc_6uyN0yw7mQvDMQ/https%3A%2F%2Fdoi.org%2F10.1007%2Fs10995-021-03157-9)

*-Thanks for the reference. We cited an earlier version of the paper and updated the reference.*

Suggestion to overcome the problems in identification:
The use of marital status as the 3rd variable makes the analysis weak since its direct association with abortion and miscarriage is unclear. Perhaps you could introduce a variable more connected to abortion and miscarriage. A possibility would be interbirth Interval (or the first birth interval for first births). The longer the last interbirth interval (or the ongoing birth interval), the more likely that missreporting is taking place. Having 3 variables measuring (somehow) the same phenomena could make the LCA interpretation more credible.

*-As we have only two indicators of abortion/miscarriage (CAPI and ACASI answers), we have to make additional assumptions to make LCA identifiable. We do not have interbirth Interval, unfortunately. We talked about the limitations of LCA when assumptions are violated in the discussion section.*

Other technical comments, although I believe the LCA and logit analysis have to be changed:

The logistic regression described in line 337 does not specify how variables have been selected. According to PLOS ONE statistical guidelines you should specify the procedure used for the identification of variables. The candidate variables should also be listed providing a rationale for the specification in the methods section. Also whether you checked for possible multicollinearity, and given the use of logistic regression, an assessment of balance. You could report it in an appendix table. It now seems that you are just putting together all those variables.

*-We explained in the discussion section that our modeling efforts were guided by past results and by the general hypothesis that women from traditional cultural backgrounds would be more likely to underreport abortions. We also used stepwise model selection procedure to find the best models. The results from these reduced models do not differ substantially from the full models. As a result, we reported the results from the full models.*

Reporting of the age-squared terms in table 4 (and 6) is wrong, showing only 0s. You should rescale so that the SE and the coefficients are shown, maybe multiplying them both by 1000. If they are all non-significant maybe it is best to remove them. Age-squared increases very much SEs given multicollinearity with age.

*-We dropped age-squared term from the models.*

There is no overall statement of the probability of underreporting using weights. They could be added to tables 3 and 5.

*-As noted in the text, the weighted percentages are similar to the unweighted percentages. We did not want to add more numbers to Tables 3 and 5 and make them more complicated than necessary.*

Reviewer #4: Dear authors
I have read your manuscript with enthusiasm and enjoyed it. I have some minor comments and hope it improves the quality of the manuscript.

1. You noted that one of the aims is to understand why abortions are so badly underreported in surveys. I think it is better to say 'to quantify the level of underreporting'. I think qualitative-type works are needed to address WHY underreporting happens

*-We implemented the change suggested by Reviewer 2.*

2. What does term 'indicator variable' mean in LCA? Do you mean independent variables offered to the model so as to predict group membership? I guess you haven't dome that, because you did not want predictors of the group membership to be independent variables in the logistic regression models. I think it is worth to address these issues in the discussion.

*-By “indicator variable,” we meant answers provided by respondents. Specifically, we used answers provided by respondents to CAPI and to ACASI separately in LCA.*

3. My understanding is that LCA can provide better estimates if we include more variables in the model. Why only 2-indicator and 3-indicator variable models are developed? Wasn't is possible to include more variables in the LCA analysis?

*-LCA can provide better estimates if we have more indicator variables. Since we only have CAPI and ACASI answers as the indicator variables, we can only fit two-indicator LCAs. The 3-indicator LCAs use CAPI and ACASI answers as well as a third indicator variable (whether or respondent reported ever being pregnant).*

4. LCA may classify some women to the over-report category. I liked the idea to force the model not to allocate any women to this category, and you showed that the results were robust. I am just curious how to interpret over-reported cases. Why some women should over-report abortion? Wasn't is possible to compare characteristics of truthful group with over-reported group?

*-We do not have direct evidence speaking to why some women would over-report abortion. Over-report could happen if a respondent, for instance, selected the wrong answer on ACASI by mistake. We could not really compare characteristics of truthful group with over-reported group given that this group is really small; over-report of abortion group ranges from 0 to 52 and over-report of miscarriage group ranges from 0 to 78.*

5. The first paragraph in the results section can be deleted

*-We dropped the paragraph.*

6. How these data are calculated? "From 6.2% (n=703) to 6.6% (n=741) of the women were assigned to the “had an abortion” class, which is a bit higher than the proportion of self-reported abortions in either CAPI or ACASI modes.

*-These numbers are from Table 2. We added the percentages to the table to make it clear how 6.2% was calculated.*

7. Tables 2 and 3 show unweighted class memberships. What does unweighted mean here? Furthermore, in line 300, you noted that "weighted figures are similar". What does weighted data mean? Which weights and how applied?

*-Unweighted means that the case counts and associated percentage were calculated without weights (using PROC FREQ). Weighted figures were calculated using survey weights and incorporating complex survey design features (using PROC SURVEYFREQ).*

8. The logistic regression outputs are not informative. I prefer to see OR, 95% CI, and P-value. Please explain on what basis variables were selected to be offered to the model? Why variable selection method, such as Backward Elimination, has not been applied? Also please report exact P-values instead of <0.10 or <0.05

-*Tables 4 and 6 would become too wide if we displayed OR, 95% CI and p-value. Instead, we added two supplemental tables (S-3 and S-4) to display the requested information and kept Tables 4 and 6 to present model results in log scale. As mention in the earlier response,* *our modeling efforts were guided by past results and by the general hypothesis that women from traditional cultural backgrounds would be more likely to underreport abortions. We also used stepwise model selection procedure to find the best models. The results from these reduced models do not differ substantially from the full models. As a result, we reported the results from the full models.*
