# Supplementary material for: Detecting underreporters of abortions and miscarriages in the national study of family growth, 2011–2015
Source: PLoS One. 2022 Aug 3;17(8):e0271288. doi: 10.1371/journal.pone.0271288 (PMC9348680; doi:10.1371/journal.pone.0271288)
Supplement: Attachment — Submitted filename: PLOS_Rebuttal_letter_06082022.docx [file pone.0271288.s006.docx]

Dear Editor,

We have revised our manuscript to address comments from reviewers. Below we explained how we addressed each comment. We hope that this revision is satisfactory to you.

Best Regard

Ting Yan and Roger Tourangeau

**Reviewers’ comments:**

**Reviewer #1:**

-First, the comments from the authors on my second point in the first review were helpful, and the revisions begin to address this issue. However, I think it would be helpful in the discussion (around lines 435-436, where revisions were made) for the authors to either provide information or use their expertise to speculate about how similar/dissimilar the underreporters they are researching (i.e., those who report on only one CAPI or ACASI) might be to other kinds of underreporters. A sentence or two in the discussion would suffice.

*-We added our speculation on differences between women who denied reporting in both modes and women who reported in ACASI but not in CAPI I in the discussion (starting with line 441).*

-Second, the clarifications provided to my comment regarding “truthful reporters” was also helpful. However, in line 158, the authors state that those who report on both CAP and ACASI are “truthful” reports of abortion, while those that report in only one are likely underreporters. It would be more accurate to call the former “consistent” reporters of abortion here. Alternatively, make explicit that you are going to assume that these consistent reporters are “truthful.” A sentence making that statement would suffice. The point about the LCA classifying “truthful” reporters is acceptable, but your argumentation should be stated clearly in the paper text. I suggest adding a brief explanation as provided in the reviewer comments in the analytic strategy section.

*-In Line 158, we changed the word ‘truthful’ to ‘consistent’ and added a sentence at the end of that paragraph clearly stating that we are assuming consistent reporters are truthful reporters (starting with line 166).*

Other Minor issues:
Lines 45-47 [There is limited evidence… and Still…] sentences seem contradictory – maybe rephrase to clarify meaning.

*-We rephrased the sentence to clarify meaning.*

Line 34 – the following citation should also be included re: Add Health: Tierney, Katherine I. 2019. “Abortion Underreporting in Add Health: Findings and Implications.” Population Research and Policy Review. doi: 10.1007/s11113-019-09511-8.

*-Thank you for the citation. We’ve added it.*

**Reviewer #2:**

1- The authors stated that abortion is more stigmatized than miscarriage.

Using the findings from the study [11] to justify this argument is better. Additionally, could you add some findings from the study [12], too?

The index that Bommaraju et al. (2016) used for abortion underreporters, shows more stigma on reporting an abortion as opposed to reporting a miscarriage.

*-Thank you for the suggestion. We’ve added one finding reported in Table 3 of Bommaraju et al. (2016), starting with line 40 .*

2- I understood that the logistic regression models using survey data only examined characteristics of women who reported abortion/miscarriage in ACASI but not in CAPI.

But, in the text (see page 15) you said:

i. “… we fit logistic regression models to compare those who underreported their abortions (i.e. reporting group 2 above) to truthful respondents (i.e. reporting group 1).”

ii. I see group 2 above on the same page: 2) those who were assigned to the class but did not report an abortion in at least one mode.

According to the statement in ii, respondents who report their abortions/miscarriages in CAPI but not in ACASI are included in Group 2). In that case, still, the logistic models were run over women who reported abortion/miscarriage in ACASI but not in CAPI? You know, these are two different subgroups of underreporters. Could you clarify it, please? You can prefer to change your statement as follows:
“… we fit logistic regression models to compare those who underreported their abortions (reported in ACASI but not in CAPI) to truthful respondents (reported in both modes).”

*- The four reporting groups described on p10 are the results of comparing women’s actual answers to the latent class they were assigned to by LCA. Both Group 1 and Group 2 were assigned to the latent class of “having an abortion.” Group 1 reported having an abortion in both modes and, as a result, they are labeled as ‘truthful reporters of abortion’. By contrast, Group 2 respondents failed to report having an abortion in either CAPI or ACASI. As a result they are labeled as ‘underreporters of abortion’.*

*We added some language to clarify the meaning of Group 2 (Lines 220-221, 224).*

3- From your point of view based on the findings, LCA may not be regarded as a success when it is compared to the method using survey data.

However, I believe that the LCA technique could be a considerable way to detect under-reporters of abortions/miscarriages, especially for different surveys that do not provide two data collection modes to gather the same information. Could you add something like that in your discussion, considering surveys that have different designs?

I believe that journal readers would like to use the LCA technique to detect underreporters of abortions/miscarriages using data coming from different surveys.

-*We’ve added a paragraph about the use of LCA for other surveys in the discussion (p24, Lines 463-467).*

4- Lastly, the LCA method did not bring much advantage in identifying of underreporters of abortions in the NSFG.

However, do you suggest the use of this method when detecting underreporters of other sensitive variables in the NSFG (such as alcohol and drug use, involuntary sex and sexual disease?)

I think, the lack of advantage of the method for abortion/miscarriage underreporters may turn when underreporting of other variables are studied.

-*We’ve added a couple sentence about the use of LCA for other variables in the discussion. (p24, Lines 463-467).*
